# Detection and Maturity Classification of Dense Small Lychees Using an Improved Kolmogorov–Arnold Network–Transformer

**DOI:** 10.3390/plants14213378

**Published:** 2025-11-04

**Authors:** Zhenpeng Zhang, Yi Wang, Shanglei Chai, Yibin Tian

**Affiliations:** College of Mechatronics and Control Engineering, Shenzhen University, Shenzhen 518060, China

**Keywords:** lychee detection, maturity classification, yield estimation, robotic harvesting, Kolmogorov–Arnold Network (KAN), Transformer

## Abstract

Lychee detection and maturity classification are crucial for yield estimation and harvesting. In densely packed lychee clusters with limited training samples, accurately determining ripeness is challenging. This paper proposes a new transformer model incorporating a Kolmogorov–Arnold Network (KAN), termed GhostResNet (GRN)–KAN–Transformer, for lychee detection and ripeness classification in dense on-tree fruit clusters. First, within the backbone, we introduce a stackable multi-layer GhostResNet module to reduce redundancy in feature extraction and improve efficiency. Next, during feature fusion, we add a large-scale layer to enhance sensitivity to small objects and to increase polling of the small-scale feature map during querying. We further propose a multi-layer cross-fusion attention (MCFA) module to achieve deeper hierarchical feature integration. Finally, in the decoding stage, we employ an improved KAN for the classification and localization heads to strengthen nonlinear mapping, enabling a better fitting to the complex distributions of categories and positions. Experiments on a public dataset demonstrate the effectiveness of GRN-KANformer. Compared with the baseline, GFLOPs and parameters of the model are reduced by 8.84% and 11.24%, respectively, while mean Average Precision (mAP) metrics mAP50 and mAP50–95 reach 94.7% and 58.4%, respectively. Thus, it lowers computational complexity while maintaining high accuracy. Comparative results against popular deep learning models, including YOLOv8n, YOLOv12n, CenterNet, and EfficientNet, further validate the superior performance of GRN-KANformer.

## 1. Introduction

**Figure 1:**
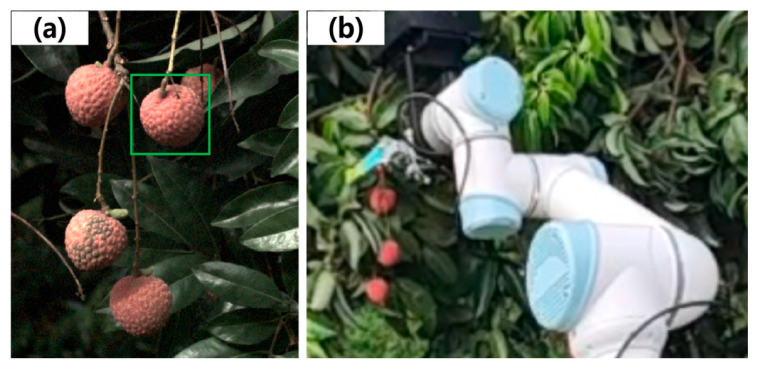
Example of lychee recognition and robotic picking: (**a**) lychee detection by computer vision; (**b**) robotic picking.

Lychee is a high-value subtropical fruit, and China accounts for about half of the world’s production [1]. During the ripening period, accurate lychee detection and maturity classification are essential for yield estimation and harvesting [2,3,4,5]. However, because lychee orchards in some regions are small and unevenly distributed, both yield estimation and harvesting face substantial challenges. Figure 1 shows an example of lychee recognition by computer vision and its robotic picking. Existing mechanical harvesters often fail to localize fruit precisely, missing small, densely clustered lychees or mistakenly grasping leaves instead, which limits yield estimation and robotic harvesting efficiency and degrades fruit quality. Therefore, developing efficient and intelligent systems for lychee localization and maturity classification is key to enhancing production efficiency and fruit quality.

As the agricultural workforce continues to shrink, manual yield estimation and harvesting in complex terrains become impractical, while vision-guided robotic methods are emerging as promising alternatives [6]. Early studies applied traditional computer vision to fruit detection. Díaz et al. [7] classified four olive varieties using contour segmentation and Mahalanobis distance-based classification, achieving fast and efficient sorting in an automated system. Bulanon et al. [8] adopted an automatic thresholding algorithm based on red-channel color-difference histograms, which improved apple segmentation accuracy and recognition rates. Some researchers segmented lychee using color thresholds [9,10], while others localized fruit by extracting fruit edges for segmentation [11]. Subsequent work introduced machine learning keypoint matching to replace purely hand-crafted methods [12]. With the rise of deep learning in the past decade, a larger body of fruit detection research leveraged learned features rather than hand-engineered descriptors, thereby mitigating the limitations of traditional methods under complex illumination and cluttered backgrounds [13,14,15]. Deep learning detectors can be broadly categorized into two families. The first comprises two-stage methods that first generate proposals and then classify and refine them, typically more accurate but slower, such as the R-CNN family [16,17,18] and SPP-Net [19]. For example, Gao et al. [20] used Faster R-CNN to detect and classify apples under different growth conditions, achieving an average classification accuracy of 87.9%. Yu et al. [21] employed Mask R-CNN to segment strawberries, compute the peduncle from the mask, and determine the picking point by extending 13–20 mm upward, with a mean localization error of 1.2 mm. The second family consists of one-stage methods that directly perform classification and regression on feature maps, represented by SSD [22] and the YOLO series [23]. Wang et al. [24] improved the YOLOv5s backbone with ShuffleNet and incorporated a CBAM module in the feature fusion stage, yielding robust accuracy across diverse scenes. Liang et al. [25] proposed a UAV-based approach for lychee ripeness classification that integrates multiple optimization modules into YOLOv8, reaching an average accuracy of 87.7%. Many of these studies emphasize lightweight modules to boost efficiency. For instance, Xie et al. [26] added a P2 small object detection layer to YOLOv8 and designed a lightweight mixed local channel attention module, improving detection of low-quality lychees in orchard environments. Other studies enhance accuracy through attention mechanisms. Peng et al. [27] added a small object detection layer for UAV imagery and introduced an efficient channel attention-enhanced YOLOv8, improving robustness and accuracy for field lychee recognition and yield estimation. In addition, many studies have applied deep learning-based object detection methods in precision agriculture. Allmendinger et al. [28] compared the performance of several state-of-the-art object detection models for real-time and accurate weed detection. The results demonstrated that the YOLO series achieved superior real-time performance, whereas Transformer-based models exhibited higher detection accuracy. Cardellicchio et al. [29] trained an improved YOLO11 model and then applied incremental transfer learning to fine-tune the model for data under different representation conditions, thereby enhancing inference speed and reducing variance.

Despite these advances, designing models that simultaneously improve efficiency and maintain accuracy for detection and maturity classification of small and densely packed fruits remains challenging [30]. Some researchers have specifically targeted field fruit image analysis. James et al. [31] developed a semantic segmentation framework for fruits using transfer learning, enabling accurate segmentation with only a small number of labeled images. Shrawne et al. [32] explored fruit detection using an Eager model with a ResNet-50 backbone under various single- and multi-class settings. Others have investigated dense and small fruit detection [33,34,35]. Tu et al. [36] proposed an improved multi-scale fast region-based CNN and used an RGB-D camera to capture images of passion fruit; the method performed well when more than 80 fruits were present in dense scenes. Lu et al. [37] introduced an effective SOD head for early-stage small fruits, yielding more accurate and stable predictions. Lin et al. [38] presented an improved YOLOv8 in which the detection head was replaced by RT-DETR and CIoU was refined with Focaler-IoU, enhancing detection of occluded small pineapples.

Recent studies show that the Kolmogorov–Arnold Network (KAN) has stronger nonlinear expressivity than multi-layer perceptions (MLPs) [39], markedly improving interpretability, computational efficiency, and adaptability for complex function approximation, as well as parameter efficiency and scalability across time series forecasting, computational biomedicine, and graph learning [40,41,42]. Zhang et al. [43] combined the adaptive nonlinearity of KAN with deep convolution operations, achieving strong performance in remote sensing segmentation with only 1% labeled data. Other work suggests that KAN may also improve detection. For example, Zhan et al. [44] fused spatial channel KAN attention into residual networks to form a Kolmogorov–Arnold Attention Network (KAAN), outperforming state-of-the-art methods on Market-1501, MSMT17, and VeRi-776 for object re-identification. Zhang et al. [45] combined KANConv with C2f to mitigate global feature loss from max pooling, thereby improving both accuracy and interpretability for defect recognition. Inspired by these studies, we propose a GhostResNet–KAN–Transformer (GRN-KANformer) network to address fruit detection and ripeness classification of densely distributed small lychees. The main contributions are as follows:

- A KAN-enhanced Transformer model (GRN-KANformer) is developed for dense small lychee detection and maturity classification in the field.
- Multiple enhancements for Transformer are proposed: a stackable, dynamic lightweight GhostResNet is incorporated; a small object detection layer is added to the hybrid encoder, enabling feature extraction from dense small lychees; an efficient channel attention mechanism is introduced for cross-level multi-scale fusion, reducing interference and strengthening lychee-specific feature extraction.
- An improved KAN module is incorporated into the detection head, enhancing detection capability without significant increases in model complexity.
- GRN-KANformer has been validated using a public lychee dataset and it outperforms multiple popular deep learning models.

## 2. Proposed Method

**Figure 2:**
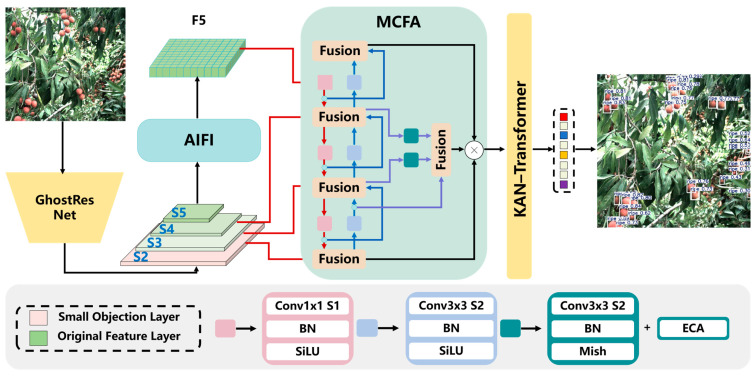
The overall architecture of the proposed GRN-KANformer. (AIFI: Attention-based Intra-scale Feature Interaction; MCFA: multi-layer cross-fusion attention; BN: batch normalization; ECA: efficient channel attention; SiLU and Mish are activation functions; details of other notations are described in RT-DETR [46]).

As shown in Figure 2, the proposed GRN-KANformer is built upon the Real-Time Detection Transformer (RT-DETR) [44], with improvements made to the three main components of the network—the backbone, neck, and head—to enhance detection performance for densely distributed small lychees. In the backbone, a lightweight GhostResNet bottleneck is employed to reduce computational complexity. In the feature encoder, we introduce a small-object feature extraction layer (S2), which is a convolutional layer followed by the S3 and S4 convolutional layers. After passing through the S5 convolutional layer, Attention-based Intra-scale Feature Interaction (AIFI) performs intra-scale interactions on S5 using a single-scale Transformer encoder to obtain F5. The original CCFF is then replaced by a Mixed Cross-layer Fusion Attention (MCFA) module, which more effectively extracts and fuses salient features while enhancing feature representation capability. A channel attention mechanism is incorporated to focus on lychees of varying maturity levels, and the final MCFA layer fuses shallow and intermediate features to better capture fine details. Finally, in the Transformer detection head, the linear head is replaced with an improved KAN, which introduces nonlinearity at minimal computational cost and helps distinguish overlapping lychees.

### 2.1. GhostResNet Block

**Figure 3:**
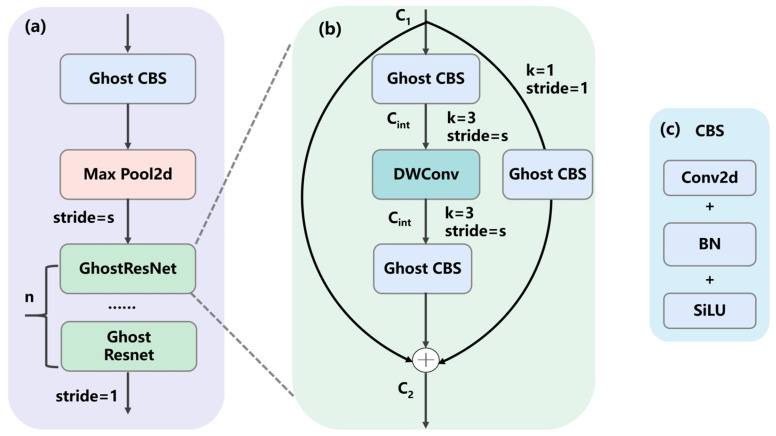
(**a**) The GhostResNet (GRN) block; (**b**) details of GRN sub-module; (**c**) details of the CBS sub-module. (s: user-defined stride; n: the number of stacked GRN layers; C_1_ and C_2_: the input and output channel counts, respectively; C_int_: the channel width after dimensionality reduction).

In on-tree lychee images, densely packed regions are typically small and often partially occluded. We combined the ideas of GhostNet [47] and ResNet [48] to design a stackable multi-layer parameterizable lightweight module named GhostResNet block (GRN), as shown in Figure 3. The first GhostCBS performs rapid downsampling and initial feature extraction, which is crucial for handling high-resolution images. Subsequent stages stack multiple GRNs to progressively deepen the network and extract increasingly abstract features.

#### 2.1.1. GhostResNet (GRN)

Mainstream CNNs exhibit substantial redundancy in intermediate feature maps when processing fruit images. We therefore design a lightweight module by combining two complementary primitives, GhostConv and depthwise separable convolution (DWConv) [49]. In GhostCBS, standard convolutions are replaced with GhostConv to reduce the number of filters required to generate feature maps, and identity mappings run in parallel with linear transformations to preserve intrinsic lychee features. Batch normalization (BN) and ReLU nonlinearity are applied after each layer, except that ReLU is omitted after the second Ghost module. Concretely, GhostCBS is used in the first layer and in the shortcut, dramatically reducing computation and network parameters; a 3 × 3 DWConv is employed in the second layer, while a residual shortcut connection aligns output dimensions and enables effective extraction of spatial features from lychee images.

#### 2.1.2. Dimensionality Reduction Hyperparameter

As shown in Figure 3, GRN includes a hyperparameter, Ratio, which allows fine control over model width and capacity. For mobile deployment, a smaller Ratio (e.g., 0.125) yields an ultra-lightweight model, whereas on servers a larger Ratio (e.g., 0.5) can improve performance. By first reducing and then restoring channel dimensionality via the Ratio setting, the intermediate channels are greatly compressed, enabling the most compute-intensive 3 × 3 DWConv to operate on a very small channel width. This flexible model scaling enlarges the effective context and makes the detector more sensitive to edge textures of small lychees.

### 2.2. Addition of the S2 Small Object Detection Layer

**Figure 4:**
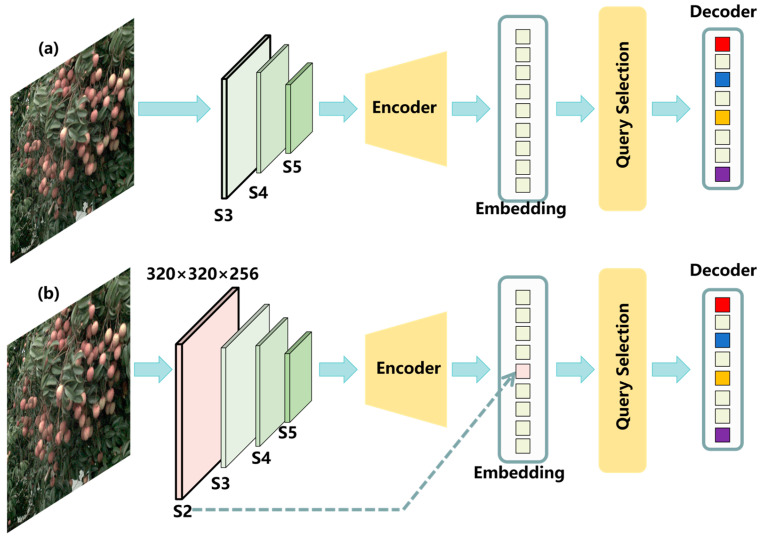
Design of the S2 small object detection layer: (**a**) original RT-DETR architecture; (**b**) RT-DETR with the added S2 small object detection layer.

Most lychees occupy only a small fraction of the image. Prior studies have shown that adding a higher-resolution feature map in the backbone improves small object detection [26,27]. Inspired by this, we modify the feature extractor in RT-DETR by adding a small object detection layer (Figure 4). Figure 4a shows the original RT-DETR feature hierarchy; Figure 3b illustrates the design of the added S2 layer. The S2 feature map is 320 × 320 × 256, providing a smaller effective receptive field and relatively richer positional cues, thereby enhancing detection of small objects. Afterwards, we augment the corresponding positional encoding with new query indices aligned to the features extracted at S2. To ease object querying, we adopt uncertainty minimization query selection [46], which explicitly models and optimizes epistemic uncertainty to capture the joint latent variables of encoder features and thus supply high-quality queries to the decoder. Specifically, the feature uncertainty integrates discrepancies between the predictive distributions of localization and classification, as expressed in Equations (1) and (2).

(1) $$U(\hat{X})=\left\|P(\hat{X})-C(\hat{X})\right\|$$

where $\;U(\hat{X})$ denotes the joint uncertainty metric, $\hat{X}$ the decoder output feature, $P(\hat{X})$ the predictive distribution of the localization branch, and $C(\hat{X})$ the predictive distribution of the classification branch.

(2) $$L=L_{box}+L_{cls}(U(\hat{X}))$$

where L is the total loss; $L_{box}$ and $L_{cls}$ are the localization branch loss and the classification loss induced by the joint uncertainty metric.

### 2.3. Multi-Layer Cross-Feature Fusion with Efficient Channel Attention

**Figure 5:**
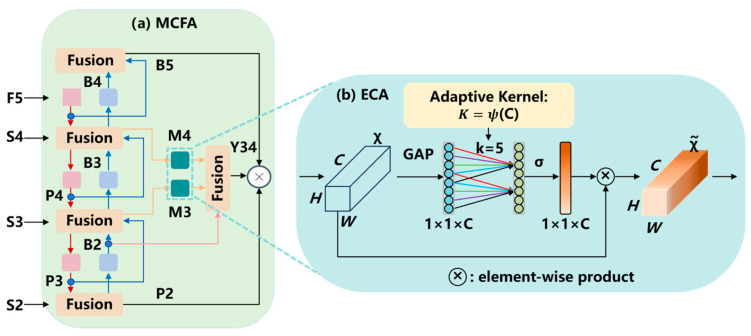
(**a**) The structure of the multi-layer cross-fusion attention (MCFA) model; (**b**) the structure of the efficient channel attention (ECA) module. (GAP: global average pooling; details of other notations are described in RT-DETR [46]).

Recent studies indicate that cross-feature fusion and attention mechanisms improve fruit detection accuracy. Chai et al. [50] enhanced YOLO for cherry tomato detection using feature fusion mechanisms, achieving significant gain over the baseline model. Yan et al. [51] addressed bagged pear detection under various occlusions by introducing multi-scale cross-modal feature fusion and a cost-sensitive classification loss, improving mAP50 by 3.6% relative to the classic YOLOv10m. Xu et al. [52] proposed a passion fruit yield estimation method that combines a density-aware attention mechanism with cross-scale feature fusion; orchard experiments showed superior identification accuracy, small fruit detection, and duplicate suppression, substantially enhancing reliability and practicality. Inspired by these findings, we design a multi-layer cross-fusion attention module that further strengthens the RT-DETR’s capacity to extract and fuse heterogeneous features (Figure 5). Layer 1 fuses deep F5, mid-level S4, shallow S3, and small object S2 features. Layer 2 continues feature extraction and performs cross-scale fusion of F5, P4, and P3 from Layer 1. Layer 3 refines B2 and B3 from Layer 2, fuses them with efficient channel attention (ECA) [53] to obtain M3 and M4, and then re-fuses with B2. The final layer unifies the mid-scale features into Y34 and forms a three-path concatenation with B5 and P2.

To further enhance high-level feature fusion for maturity classification, we employ ECA in the third fusion layer. As shown in Figure 5b, it captures local cross-channel interactions by considering each channel and its neighboring channels, an approach proven to be both efficient and effective. It uses a 1D convolution to avoid dimensionality reduction, providing an efficient mechanism for cross-channel interaction.

### 2.4. KAN-Enhanced Detection Head

On-tree lychees often occur in clusters with severe occlusions, which demands stronger nonlinearity for both detection and classification. Recent studies have shown that combining an improved KAN with a Transformer yields promising results [54]. Specifically, three strategies are adopted to overcome the large parameter count and low computational efficiency of conventional KANs: (a) using rational functions as basis functions; (b) sharing function coefficients and basis functions across groups; and (c) a variance-preserving initialization to keep activation variance consistent across layers. The main formulations of the improved KAN are as follows:

(3) $$x_{0}^{\left(i\right)}=MSA(LN(x_{i-1}))+x_{i-1},\;i\;=1,\ldots L$$

(4) $$x_{i}=KAN(LN(x_{0}^{\left(i\right)}))+x_{0}^{\left(i\right)}$$

where $x_{i}$ is the ith output feature sequence, LN denotes layer normalization, and MSA multi-head self-attention.

**Figure 6:**
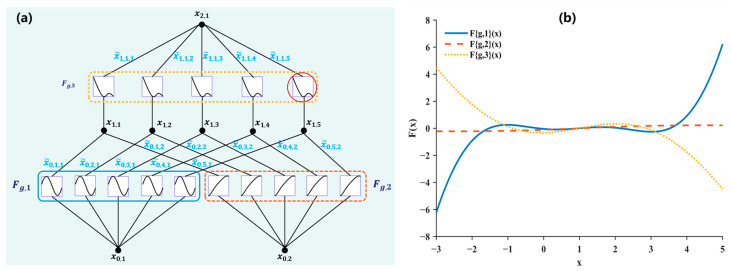
(**a**) Illustration of the modified KAN architecture; (**b**) an example of individual activation function, as indicated by the red circle in (**a**).

As shown in Figure 6, the improved KAN architecture uses three differently colored dashed boxes to denote distinct parameter groups. Within each group, all edges (i.e., connections from input channels to output channels) share a single learnable nonlinear basis function, $F_{g}$, rather than assigning a separate set of functions to every edge as in the original KAN. Moreover, all input channels within the same group (i.e., multiple inputs) share the same $F_{g}$ across all outgoing edges. The right panel of Figure 5 illustrates one exemplar activation profile for a group. The rational polynomial basis functions are given as follows:

(5) $$\emptyset (x)=\omega F(x)=\omega \frac{a_{0}+a_{1}x+\cdots +a_{m}x^{m}}{1+|b_{1}x+\cdots +b_{n}x^{n}}$$

where ω is the scaling factor, and $a_{i}$ and $b_{j}$ denote the polynomial coefficients, which are learned end-to-end.

Following the idea of replacing MLPs with a nonlinear alternative, we substitute the linear layers in the detection head with a KANLinear operator. Because these linear layers govern both bounding box regression and class prediction, replacing them with KANLinear promotes a more unified vector representation and better fits complex nonlinear distributions, yielding more accurate lychee bounding boxes and maturity classifications.

## 3. Experimental Results

To validate the effectiveness of the proposed GRN-KANformer and assess its performance, we conducted experiments using a public lychee dataset characterized by small and densely distributed fruits. We also compare it against several state-of-the-art deep learning models (RT-DETR, YOLOv5, YOLOv8, YOLOv12, CenterNet, and EfficientNet). Performance was comprehensively evaluated in terms of precision, recall, computational complexity (GFLOPs), and model parameter size.

### 3.1. Dataset and Experimental Settings

In the lychee harvesting literature, most studies are based on single-fruit detection and classification [4,12,26]. The dataset originated from our previous work; details of image acquisition are provided in [55]. Images have a resolution of 1024 × 1280 and are annotated with three maturity levels: unripe, semi-ripe, and ripe. As illustrated in Figure 6a, red boxes indicate the definition of small lychee. We computed bounding box pixel areas and defined a small lychee as one with an area < 1600 px (≈0.12% of the full image). We defined dense scenes as those in which lychee annotations constitute > 30% of all annotations in an image. From the on-tree lychee dataset, we selected 225 images that meet these criteria, forming our dense and small lychee image subset.

**Figure 7:**
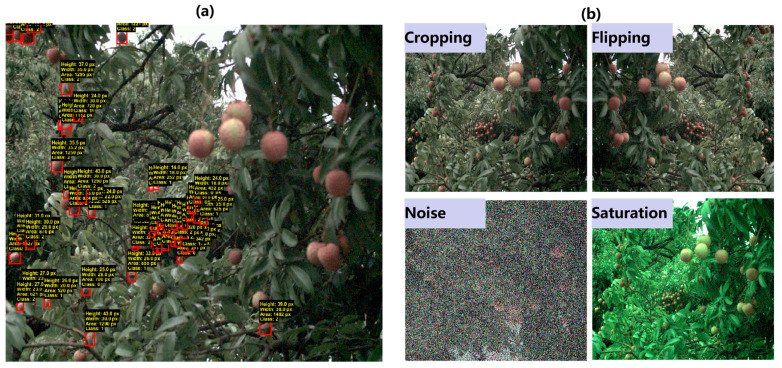
(**a**) Illustration of small lychees (those in red bounding boxes) defined in the current study; (**b**) examples of different image augmentation methods.

The generalization ability of a dataset greatly impacts the performance of the trained deep learning model. To achieve good generalization, the validation error must continue to decrease as the training error decreases. Data augmentation is a powerful method to accomplish this. It generates more comprehensive data samples, thereby minimizing the gap between the training set, validation set, and test set [56]. Geometric transformations of images often preserve annotations because they only alter the positions of key features, making the transformation functions relatively safe [57]. In this work, we applied scaling, cropping, random rotation, horizontal and vertical flipping, and center cropping. Real-world data are rarely perfect [58]; when neural networks are evaluated using real data, noise can degrade accuracy and lead to poor generalization. Robustness can be improved by augmenting the data with various types of noise. In our case, Gaussian, Salt and Pepper, and Poisson noises, which are common in real images, were used to enhance the dataset. Since images are typically encoded as tensors with three dimensions—height, width, and channel—performing data augmentation in the color channel space is another effective strategy. Through basic matrix operations, RGB values can be scaled to increase or decrease image brightness [59]. Random brightness, contrast, and saturation adjustments were applied to generate new images distinct from the originals. Visualization examples of the augmented data are shown in Figure 7b.

After data augmentation, the dataset comprises a total of 1125 images with 15,344 annotations. Following a 9:1:1 split ratio, it is divided into a training set (900 images, 12,448 annotations), a validation set (110 images, 1460 annotations), and a test set (115 images, 1436 annotations).

The proposed algorithm was developed based on the Ultralytics library [60] (version 8.3.179), Python (version 3.10.18), and PyTorch (version 2.8.0). All experiments were conducted on a workstation equipped with an Intel i7 CPU (Santa Clara, CA, USA) and an NVIDIA GeForce RTX 4090 GPU (Santa Clara, CA, USA). During training, all images were resized to 640 × 640 pixels. The batch size was set to 16, and the training was performed for 1000 epochs with an early stopping patience of 100 epochs (training terminated if no significant improvement was observed). The initial learning rate was set to 0.01 and decayed to 1% of its initial value. The Stochastic Gradient Descent (SGD) [45] was used as the optimizer with a momentum of 0.937. The weight decay coefficient was set to 0.0005 to regularize the model parameters. All other hyperparameters were kept at their default values.

### 3.2. Evaluation Metrics

We use per-class and overall precision (P), recall (R), and mean Average Precision (mAP50) as the basic metrics. We also report mAP50–95, i.e., the average mAP over IoU thresholds from 0.50 to 0.95 in steps of 0.05, which provides a more comprehensive assessment. The formulas for per-class P/R/AP/mAP are given in Equations (6) and (7), and those for overall P/R/AP/mAP are given in Equations (8) and (9).

(6) $$P_{i}=\frac{{TP}_{i}}{{TP}_{i}+{FP}_{i}},R_{i}=\frac{{TP}_{i}}{{TP}_{i}+{FN}_{i}}$$

(7) $$AP=\sum_{i=1}^{N}(R_{i}-R_{i-1})P_{i},{mAP}_{50,i}=AP(IoU\geq 0.5),{mAP}_{50-95,i}=AP(0.95\geq IoU\geq 0.5)$$

where index i indicates the class; TP, FP, and FN represent the true positives, false positives, and false negatives; and $R_{i}$ and $R_{i-1}$ are consecutive recall levels.

(8) $$P=\frac{1}{N}\sum_{i=1}^{N}P_{i},R=\frac{1}{N}\sum_{i=1}^{N}R_{i}$$

(9) $${mAP}_{50}=\frac{1}{N}\sum_{i=1}^{N}{mAP}_{50,i},{mAP}_{50-95}=\frac{1}{N}\sum_{i=1}^{N}{mAP}_{50-95,i}$$

where N denotes the number of maturity levels.

### 3.3. Selecting the Dimensionality Reduction Hyperparameter

As discussed above, the GRN block includes a hyperparameter Ratio that controls the channel widths of the second and third layers within the block, thereby governing model size and computational cost. We conducted a hyperparameter study on Ratio. The base model is RT-DETR–ResNet50 (DETR-Res50). Its five ResNet backbone stages are replaced with GRN blocks, using the same Ratio in all stages. We evaluate three settings: 0.125, 0.25, and 0.5. The comparative results are summarized in Table 1.

All new configurations yield fewer parameters than the original DETR-Res50, with the maximum and minimum reductions being 46.37% and 29.32%, respectively, confirming the effectiveness of GRN as a lightweight backbone block. Aggregating across overall and per-class metrics, Ratio = 0.25 (GRN_0.25_) attains the largest number of best scores and is therefore adopted as the default setting for subsequent experiments.

### 3.4. Ablation Experiments

To assess the contribution of each component in GRN-KANformer, we conducted a series of ablation studies by removing one or more modules from the full model. The baseline is the DETR-Res50, and all ablations are performed to incrementally add components to the baseline to verify the effectiveness of each module. The ablation configurations are summarized in Table 2, and the evaluation follows the overall (all-class) metrics defined in Equations (8) and (9). Specifically, we begin with the baseline; then, we add, in turn, the S2 small object detection layer, the MCFA module, the GRN_0.25_ lightweight module, and the KAN-based improved detection head.

The baseline achieves a precision of 0.760, a recall of 0.816, and mAPs of 0.823 (mAP_50_l) and 0.512 (mAP_50–95_). After adding the S2 small-object module (+S2), GFLOPs and parameters increase by 6.13% and 0.67%, respectively, yet both precision and recall improve substantially; mAP_50_ and mAP_50–95_ rise to 0.832 (≈+1.09%) and 0.526 (≈+2.73%), indicating more accurate bounding box localization. Incorporating the MCFA further lifts recall to 0.850, with mAP_50_ and mAP_50-95_ reaching 0.893 and 0.562, i.e., gains of approximately 8.51% and 9.77% over the baseline. Stacking five GRN (0.25) lightweight blocks in the backbone reduces GFLOPs and parameters by 36.78% and 40.69%, confirming its positive effect on model compactness. With the KANLinear detection head added, both localization and classification improve markedly: precision and recall reach 0.863 and 0.885 (relative gains of 13.55% and 8.46% vs. baseline), while mAP_50_ and mAP_50-95_ attain 0.947 and 0.584 (improvements of 15.07% and 14.06%). Meanwhile, GFLOPs and parameters drop to 114.5 and 37.228, reductions of 8.84% and 11.24%. Overall, the results show a favorable balance between improved accuracy and the lightweight design.

**Figure 8:**
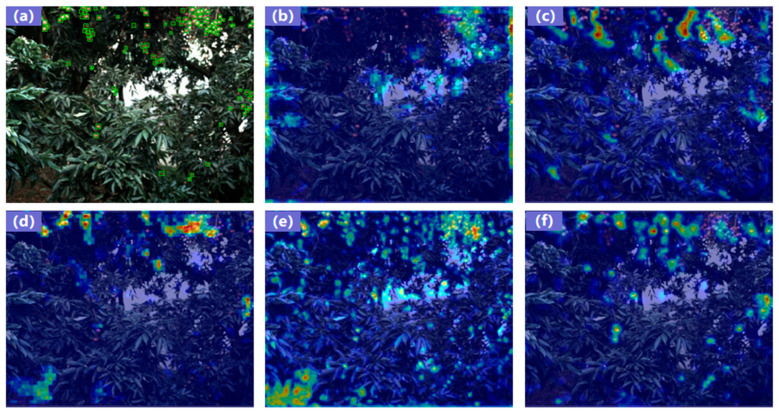
Comparison of heatmaps for ablation experiments: (**a**) ground truth; and heatmaps for (**b**) baseline (DETR-Res50), Grad-CAM extracted from the 22nd RepC3 layer; (**c**) +S2, Grad-CAM extracted from the 1st Conv layer; (**d**) +MCFA, Grad-CAM extracted from the 21st, 30th, and 36th RepC3 layers; (**e**) +GRN0.25, Grad-CAM extracted from the 4th GRN layer; (**f**) +KAN (full model), Grad-CAM extracted from the 1st S2 layer and the 21st, 30th, and 36th RepC3 layers.

To analyze which image regions are influenced by each component, we conducted a Gradient-weighted Class Activation Mapping (Grad-CAM) heatmap comparison for the ablation settings (Figure 8) [61]. Grad-CAM visualizes the regions a CNN attends to when making a specific decision by producing a heatmap that highlights areas most critical to the prediction. The baseline exhibits focused attention on most targets but also allocates attention to some non-lychee regions. After adding MCFA, S2, and KAN, the attention over small lychees becomes progressively more accurate and concentrated. In particular, the S2 and KAN modules sharpen the attention distribution on small fruits, yielding the most optimal focus across targets and notably improving detection under heavy occlusion.

GRN-KANformer is compared with other popular deep learning model, DETR-Res50, YOLOv5n [62], YOLOv8n [63], YOLOv12n [64], Fast R-CNN [17], CenterNet [65], and EfficientNet [66]. The quantitative results are summarized in Table 3.

### 3.5. Comparison with State-of-the-Art Deep Learning Models

**Figure 9:**
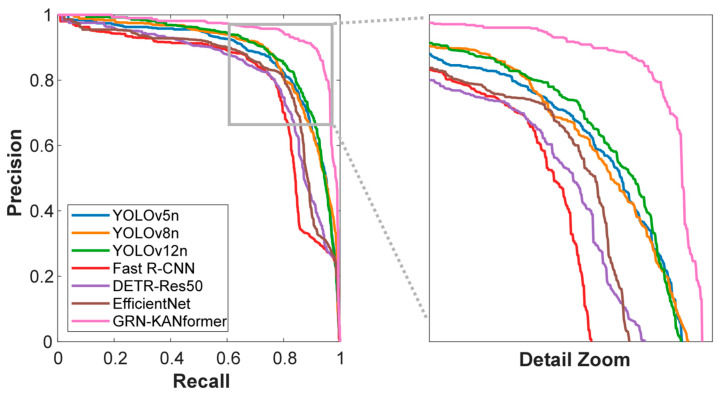
Precision–recall curves of different deep learning models.

GRN-KANformer delivers substantial gains over the baseline and the classic two-stage detector (Fast R-CNN) on nearly all key metrics. Compared to Fast R-CNN, it improves precision, recall, mAP_50_, and mAP_50–95_ by 19.20%, 11.74%, 17.93%, and 15.42%, respectively. Even against the second-best models in each metric, it maintains a favorable margin: compared with YOLO12n, precision and mAP_50_ increase by 0.35% and 6.29%, respectively; compared to CenterNet, the gain in recall is 4.61%; and compared to YOLOv5n, mAP_50–95_ increases by 5.61%. In terms of computational efficiency, compared with Fast R-CNN and DETR-Res50, it achieves higher accuracy while reducing parameters by 8.8–28.9% and GFLOPs by 10–11.2%, indicating a good accuracy–efficiency balance. Compared to ultra-lightweight YOLO variants and EfficientNet, it trades a larger model size and computing cost for 5–12% absolute accuracy gains. Figure 9 compares the precision–recall curves at IoU = 0.50 for the models.

**Figure 10:**
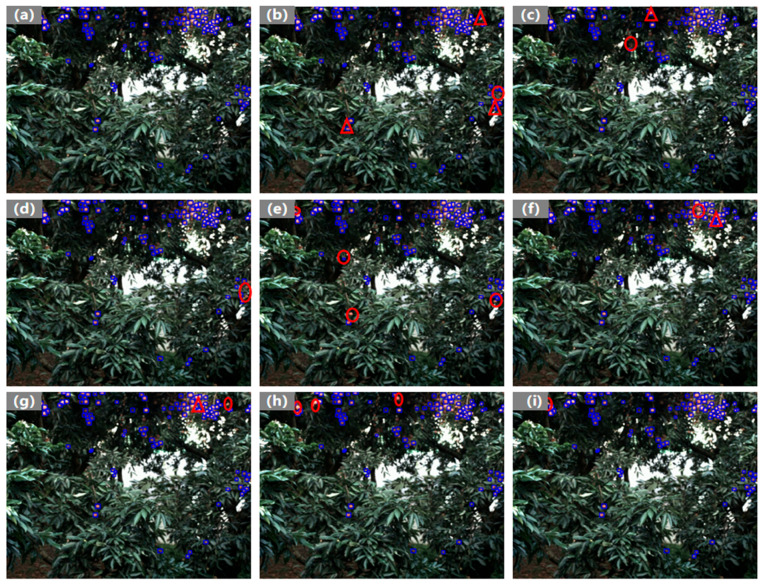
Visualization of lychee detection by different deep learning models: (**a**) ground truth; (**b**–**i**) results from YOLOv5n, YOLOv8n, YOLOv12n, Fast R-CNN, CenterNet, EfficientNet, DETR-Res50, and GRN-KANformer, respectively. Red circles mark missed detections, and red triangles indicate inaccurate localization.

Figure 10 visualizes detection results in dense small lychee scenes. Red circles mark missed detections, and red triangles indicate inaccurate localization. The compared methods exhibit varying degrees of incomplete detection, frequently missing small or overlapping objects and yielding suboptimal boundary. By contrast, GRN-KANformer detects dense small lychees more accurately, achieves notably higher recall for small and partially occluded fruits, and produces predicted boxes with high overlap to the ground truth (GT).

**Figure 11:**
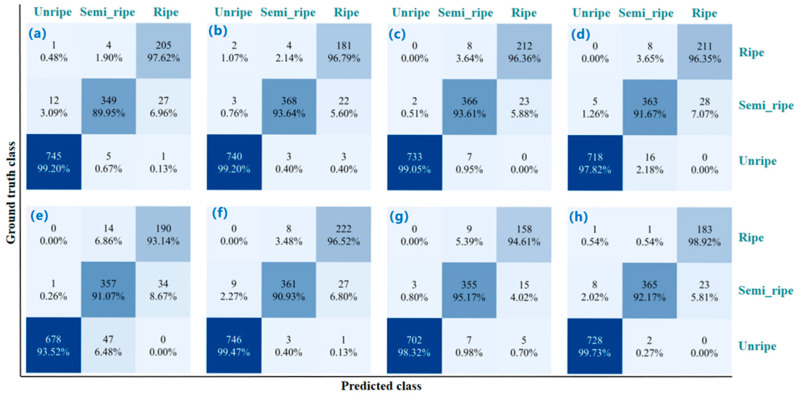
Confusion matrices of maturity classification by different deep learning models: (**a**–**h**) YOLOv5n, YOLOv8n, YOLOv12n, Fast R-CNN, CenterNet, EfficientNet, DETR-Res50, and GRN-KANformer, respectively.

Figure 11 shows the per-class maturity classification results. Although the YOLO family is generally robust, it still exhibits noticeable bias between semi-ripe and ripe, with visible misclassifications as ripe. The results from DETR-Res50 suggests that query-based decoding helps with fine-grained boundaries, yet extremely similar appearances still require priors or strengthened local attention. CenterNet, EfficientNet, and Fast R-CNN display more pronounced one-way confusion for semi-ripe, indicating limited capture of multi-scale contextual and small lychee details. By contrast, GRN-KANformer shows negligible misses for unripe fruits, and its correct semi-ripe and ripe classifications rank the highest, reflecting stronger boundary discrimination and fewer misclassifications between adjacent ripeness levels.

To further evaluate the performance of the proposed GRN-KANformer, we conducted comparative experiments with models of similar parameter scales, including YOLOv8-s/m and YOLOv12-s/m/l. Each model was trained three times with different random seeds, and the mean and standard deviation of all metrics were reported (see Table 4 and Figure 9). GRN-KANformer achieved the best performance across all indicators—precision (0.867 ± 0.015), recall (0.883 ± 0.002), mAP_50_ (0.925 ± 0.002), and mAP_50–95_ (0.585 ± 0.003), surpassing the best YOLO baselines (YOLOv12-m, mAP_50_ = 0.890; YOLOv8-m, mAP_50–95_ = 0.559) by approximately 3.9% and 4.7%, respectively. With its higher computational complexity (114.5 GFLOPs, 37.23 M parameters) compared to YOLOv8-m (78.7 GFLOPs, 25.84 M parameters), GRN-KANformer demonstrates a good performance–complexity trade-off, exhibiting consistent convergence, low metric variance (±0.003–0.015), and strong robustness across experiments. These results confirm its enhanced accuracy, stability, and generalization capability for lychee detection tasks.

## 4. Discussion

Lychee detection and maturity classification are prerequisites for unmanned yield estimation and robotic harvesting. Prior work has addressed small fruit detection to some extent [25,33,35,37]. In this study, we modified KAN and incorporated it into a Transformer, along with other improvements, to form GRN-KANformer for the same purpose. Against seven popular deep learning models, it achieves the best results on four metrics: precision, recall, mAP_50_, and mAP_50–95_. Among the eight models, it ranks sixth in parameter size and fifth in GFLOPs, indicating that the accuracy gains come at some cost to model complexity and computational cost, though it achieves significant improvement compared to the baseline DETR-Res50. For the model to run on an edge-computing device such as in harvesting robots, this may still need further work for practical deployment.

Our results also reveal a two-sided nature of KAN: if used improperly, KAN can increase representational capacity but also inflate parameters, consistent with prior findings [41,43]. We observed that replacing MLPs with a traditional KAN in the feature extraction or fusion stages increased parameters and degraded performance, specifically when substituting the MLPs in AIFI and in the decoder FFN. Instead, replacing only the linear layers in the detection head yielded better results on small lychee images than other models. Nevertheless, performance may decline in unseen scenarios (e.g., fruit varieties absent from the training set).

It should be noted that lychees also tend to grow in bunches, but the clustering is not as obvious as grapes or cherry tomatoes. Depending on the applications, it may be desirable to detect lychees and the maturity level individually, as performed in this study in some cases; however, in other cases, it may be better to detect them and classify their maturity levels in bunches. We do not have specific data on this, but we suspect that how clustered lychees are depends on the varieties. In the case of cherry tomatoes, both approaches have been explored [67,68], while for grapes, usually detection and harvesting are carried out in bunches [69]. However, the maturity level definition is likely to be less objective in bunches than in individual fruits.

Future work will explore the following: (1) expanding and diversifying the dataset to cover lychees of different sizes, cultivars, and environmental conditions, thereby enhancing generalization and reducing overfitting, including detection and classification in both individual fruit and bunches; (2) extending the framework to other fruits (e.g., strawberries and smaller blueberries) to broaden applicability in precision agriculture; and (3) developing an integrated multi-fruit detector to reduce data collection and annotation overhead across fruit types, improve model generality, and enable more robust agricultural applications.

## 5. Conclusions

This study presents GRN-KANformer, a KAN-integrating transformer tailored to fruit detection and maturity classification for small and dense lychees. By stacking purpose-designed lightweight modules, adding a small object detection layer, employing multi-layer cross-fusion attention in the neck for feature extraction and integration, and enhancing the detection head with KAN, the proposed framework improves both efficiency and robustness for lychee detection and classification under limited data. Comprehensive comparative experiments validate the superiority of the model and highlight its potential for practical deployment in precision agriculture.

## Figures and Tables

**Table 1:** Comparison of models with varying Ratio values. Bold text represents the best result for the metric.

Maturity	Metric	Ratio			
		1 (Baseline)	0.125	0.25	0.5
All	Precision	0.760	0.812	**0.817**	0.801
	Recall	0.816	0.791	**0.794**	0.786
	mAP_50_	**0.823**	0.805	0.821	0.801
	mAP_50-95_	0.512	0.517	**0.528**	0.508
	GFLOPs	125.600	**71.900**	79.4	94.3
	Paras (M)	41.941	**22.489**	24.873	29.641
Unripe	Precision	0.749	**0.807**	0.803	0.778
	Recall	0.968	**0.966**	0.957	0.969
	mAP_50_	0.925	0.906	**0.923**	0.933
	mAP_50-95_	0.598	0.607	**0.618**	0.618
Semi_ripe	Precision	0.809	0.838	**0.847**	0.839
	Recall	0.704	**0.719**	0.714	0.720
	mAP_50_	0.762	0.768	**0.771**	0.752
	mAP_50-95_	0.461	0.743	**0.479**	0.470
Ripe	Precision	0.723	0.791	**0.800**	0.786
	Recall	**0.777**	0.690	0.711	0.671
	mAP_50_	**0.782**	0.743	0.768	0.720
	mAP_50-95_	0.478	0.466	**0.488**	0.436

**Table 2:** Ablation results. Best results are in **bold**; second-best are underlined. (Paras: parameters.)

Model	Precision	Recall	mAP_50_	mAP_50–95_	GFLOPs	Paras (M)
Baseline	0.760	0.816	0.823	0.512	125.6	41.940
+S2	0.808	0.823	0.832	0.526	133.3	42.220
+MCFA	0.845	0.850	0.893	0.562	126.7	42.137
+GRN_0.25_	0.827	0.864	0.881	0.548	**79.4**	**24.873**
+KAN (Full)	**0.863**	**0.885**	**0.947**	**0.584**	114.5	37.228

**Table 3:** Comparison with other models. P: precision; R: recall; Paras: parameters. Best results are in **bold**; second-best are underlined.

Model	Precision	Recall	mAP_50_	mAP_50–95_	GFLOPs	Paras (M)
YOLOv5n	0.819	0.807	0.875	0.553	7.100	**2.503**
YOLOv8n	0.852	0.809	0.882	0.548	8.100	3.006
YOLOv12n	0.860	0.810	0.891	0.557	**6.300**	2.557
Fast R-CNN	0.724	0.792	0.803	0.506	161.074	41.358
CenterNet	0.810	0.846	0.861	0.538	150.215	32.116
EfficientNet	0.832	0.807	0.844	0.521	91.932	18.389
DETR-Res50	0.760	0.816	0.823	0.512	125.600	41.940
GRN-KANformer	**0.863**	**0.885**	**0.947**	**0.584**	114.500	37.228

**Table 4:** Experimental results of several deep learning models with comparable parameter scales under three different random seeds. The values are presented as mean ± standard deviation. **Bold** values represent the best results.

Model	Precision	Recall	mAP_50_	mAP_50–95_	GFLOPs	Paras (M)
YOLOv8s	0.825 ± 0.022	0.814 ± 0.035	0.881 ± 0.003	0.556 ± 0.003	28.400	11.126
YOLOv8m	0.857 ± 0.018	0.801 ± 0.017	0.887 ± 0.004	0.559 ± 0.003	78.7	25.841
YOLOv12s	0.821 ± 0.004	0.846 ± 0.012	0.882 ± 0.003	0.558 ± 0.003	**21.2**	**9.232**
YOLOv12m	0.817 ± 0.032	0.850 ± 0.006	0.890 ± 0.004	0.557 ± 0.003	67.1	20.107
YOLOv12l	0.842 ± 0.010	0.818 ± 0.025	0.880 ± 0.003	0.548 ± 0.004	88.6	26.341
GRN-KANformer	**0.867 ± 0.015**	**0.883 ± 0.002**	**0.925 ± 0.002**	**0.585 ± 0.003**	114.500	37.228

## Data Availability

The data used in this study are publicly available, as described in reference [53], and the link is https://data.mendeley.com/datasets/6svnttj9g4/1 (accessed on 2 November 2025). The code for this study is made public at https://github.com/SeiriosLab/Lychee/tree/main/Code (accessed on 2 November 2025).
